# Balancing pragmatism, norms and power relations: a qualitative study among post-abortion intrauterine device users in central Uganda

**DOI:** 10.1080/26410397.2025.2604886

**Published:** 2026-01-02

**Authors:** Emelie Looft-Trägårdh, Herbert Kayiga, Othman Kakaire, Josaphat Byamugisha, Kristina Gemzell-Danielsson, Amanda Cleeve

**Affiliations:** a PhD student, Department of Women and Children’s Health, Karolinska Institutet, Stockholm, Sweden.; b Senior Lecturer, Department of Obstetrics and Gynecology, Makerere University College of Health Sciences, Kampala, Uganda; c Associate Professor, Department of Obstetrics and Gynecology, Makerere University College of Health Sciences, Kampala, Uganda; d Professor, Department of Obstetrics and Gynecology, Makerere University College of Health Sciences, Kampala, Uganda; e Professor, Department of Women and Children’s Health, Karolinska Institutet, Karolinska University Hospital, Stockholm, Sweden; f Associate Professor, Department of Women and Children’s Health, Karolinska Institutet; Associate Professor, Department of Global Public Health, Karolinska Institutet, Karolinska University Hospital, Stockholm, Sweden

**Keywords:** intrauterine device, post-abortion care, contraceptives, Uganda, qualitative research, reproductive decision-making, socio-cultural norms, stigma, gender norms

## Abstract

While almost half of all pregnancies in Uganda are unintended, the use of post-abortion intrauterine devices (IUDs) remains low. In this study, we explored how women in Uganda with current or recent post-abortion IUD use navigated socio-cultural factors, norms and power relations, and overcame challenges that often hinder contraceptive use in this context. Between January and August 2023, we conducted a qualitative study at four health facilities in central Uganda. The study included 24 in-depth interviews with women aged 19 years and above who had used an IUD following treatment for incomplete abortion (spontaneous or induced). The data were transcribed and coded in NVivo and analysed using reflexive thematic analysis. Respondents justified their IUD use through a combination of pragmatic reasoning, personal autonomy and economic considerations. The right to bodily integrity, alongside practical reflections on financial constraints, gender roles and societal expectations, emerged as important considerations. Compassionate post-abortion contraceptive counselling enhanced confidence in their decision and dispelled myths and misconceptions. Concealed IUD use enabled participants to pursue their reproductive goals, while evading influence from partners, peers, or social norms that discourage use. Our findings highlight the potential of post-abortion contraceptive counselling in supporting women’s choices, and the role of concealed IUD use in realising reproductive goals. Integrating arguments around bodily autonomy in post-abortion contraceptive counselling and advocacy and supporting overt and covert use may be important strategies to strengthen sexual and reproductive health and rights in this setting.

## Plain language summary

The intrauterine device (IUD), commonly known as “the coil”, is very effective in preventing pregnancies, but many barriers to use exist in Uganda and the current use is low. In this study, we explored how women in Uganda, who had chosen an IUD after receiving treatment for abortion complications (following induced or spontaneous abortion), overcame obstacles and challenges related to the use, such as gender norms, power relations and socio-cultural factors. We interviewed 24 women from rural and urban areas in Uganda. Our analysis revealed that women opted to use an IUD following incomplete abortion, based on the belief that they had the right to decide about matters related to their body, combined with pragmatic thinking around relationship dynamics, financial circumstances, and gender roles and responsibilities. Compassionate post-abortion contraceptive counselling helped women in their decision-making around IUD use and dispelled misconceptions. Keeping their IUD use a secret enabled women to evade influence from partners, friends or social norms. Initiatives to improve access to quality post-abortion contraceptive counselling and strengthening women´s self-efficacy to use effective contraception, be it overt or covert, could be important strategies when aiming to enhance women´s reproductive health and rights in Uganda.

## Résumé

Alors que près de la moitié des grossesses en Ouganda sont non désirées, le recours au dispositif intra-utérin (DIU) après un avortement demeure faible. Dans cette étude, nous avons examiné comment les Ougandaises utilisant ou ayant utilisé récemment un DIU après un avortement ont géré les facteurs socioculturels, les normes et les relations de pouvoir, et ont surmonté les difficultés qui entravent souvent l’utilisation de contraceptifs dans ce contexte. Entre janvier et août 2023, nous avons réalisé une étude qualitative dans quatre établissements de santé en Ouganda central. L’étude comprenait 24 entretiens approfondis avec des femmes âgées de 19 ans et plus ayant utilisé un DIU après un traitement pour avortement incomplet (spontané ou provoqué). Les données ont été transcrites et codées sur NVivo, puis examinées à l’aide d’une analyse thématique réflexive. Les participantes ont justifié leur utilisation du DIU par une combinaison de raisonnement pragmatique, d’autonomie personnelle et de considérations économiques. Le droit à l’intégrité physique, ainsi que des réflexions pratiques sur les contraintes financières, les rôles de genre et les attentes de la société sont apparus comme des considérations importantes. Un conseil bienveillant en matière de contraception après un avortement a renforcé la confiance des femmes dans leur décision et a dissipé les mythes et les idées erronées. L’utilisation dissimulée d’un stérilet a permis aux participantes de poursuivre leurs objectifs en matière de procréation, tout en s’affranchissant de l’influence de leurs partenaires, de leurs pairs ou des normes sociales qui en découragent l’emploi. Nos résultats mettent en lumière le potentiel du conseil en matière de contraception après un avortement pour soutenir les choix des femmes ainsi que le rôle de l’utilisation dissimulée d’un stérilet dans la réalisation de ces objectifs. Intégrer les arguments relatifs à l’autonomie physique dans le conseil et le plaidoyer en matière de contraception après un avortement, et soutenir l’utilisation, qu’elle soit visible ou dissimulée, pourrait constituer d’importantes stratégies pour renforcer la santé et les droits sexuels et reproductifs dans ce contexte.

## Resumen

Aunque casi la mitad de todos los embarazos en Uganda no son intencionales, el uso de dispositivos intrauterinos (DIU) postaborto continúa siendo bajo. En este estudio, exploramos cómo las mujeres en Uganda con uso actual o reciente de DIU postaborto navegaron los factores socioculturales, las normas y las relaciones de poder, y superaron los retos que a menudo obstaculizan el uso de métodos anticonceptivos en este contexto. Entre enero y agosto de 2023, realizamos un estudio cualitativo en cuatro establecimientos de salud en Uganda central. El estudio consistió en 24 entrevistas a profundidad con mujeres de 19 años o más que habían utilizado un DIU después del tratamiento de aborto incompleto (espontáneo o inducido). Los datos fueron transcritos y codificados en NVivo y analizados utilizando análisis temático reflexivo. Las personas entrevistadas justificaron su uso de DIU con una combinación de razonamiento pragmático, autonomía personal y consideraciones económicas. Entre las consideraciones importantes figuraban el derecho a la integridad corporal, reflexiones prácticas sobre limitaciones financieras, roles de género y expectativas de la sociedad. La consejería anticonceptiva compasiva postaborto mejoró la confianza de las mujeres en su decisión y disipó los mitos y conceptos erróneos. El uso oculto del DIU les permitió a las participantes luchar por sus objetivos reproductivos, a la vez que evadían la influencia de sus parejas, pares o normas sociales que desalientan el uso. Nuestros hallazgos ponen de relieve el potencial de la consejería anticonceptiva postaborto para apoyar las decisiones de las mujeres, y el papel del uso oculto del DIU en realizar los objetivos reproductivos. La integración de los argumentos sobre la autonomía corporal en la consejería anticonceptiva postaborto y en la incidencia política y el apoyo del uso abierto y encubierto podrían ser estrategias importantes para reforzar la salud y los derechos sexuales y reproductivos en este ámbito.

## Background

Despite a steady decrease in fertility in Uganda since the 1980s, the fertility rate is still one of the highest in the world, with 5.2 children born per woman.^1^ While modern contraceptive use in Uganda has been increasing, it remains low at 35% and 47% for married and unmarried sexually active women, respectively. Research among female adolescents has revealed prevalence rates below 10%.^2^

The high unmet need for contraception of 28%^3^ is driven by several multilevel factors related to supply and demand. These include lack of modern methods available, equipment, trained staff and guidelines on post-abortion contraceptive counselling, as well as long distances to health clinics, fear of side effects and contraceptive misconceptions among both men and women.^4–7^ Further, socio-cultural and gender-related factors – such as norms promoting large families, gender power imbalances and male dominance in reproductive decision-making – contribute to the sustained low use of contraception.^8^ Several studies show how women carry the responsibility for their children but have little decision-making and financial power in the realm of traditional norms and patriarchal values, which obstructed their contraceptive use.^9,10^

Contraceptive use enables women and couples to influence the timing and number of births. It contributes to improved health outcomes by reducing the incidence of unintended pregnancies and associated risks^11,12^ while also improving gender equality, educational attainment and socioeconomic development.^12–14^

In Uganda, induced abortion is permitted to preserve the life and health of a woman. However, contradictions between the penal code, the constitution, and reproductive health policy – combined with pervasive stigma surrounding abortion – make it extremely difficult to access safe, legal abortion services.^15,16^ Around 46% of all pregnancies in Uganda are unintended – a rate higher than in other East African countries^17^ – which significantly contributes to unsafe abortion and related maternal mortality and morbidity.^4^

Post-abortion care (PAC) encompasses medical treatment of complications following both spontaneous and induced abortions and should be universally accessible and of good-quality, irrespective of the prevailing legal context.^18^ However, research shows that this is not always the case in Uganda^19^ with stigma around induced abortion playing a critical role by extending to women seeking PAC and providers of PAC, contributing to self-stigma, delayed care seeking and sometimes to poor quality of care.^20^

A key component of PAC is post-abortion contraceptive counselling, which plays a vital role in enhancing access to contraception and reducing the risk of unintended pregnancy.^21^ Research from Uganda indicates that such counselling is not always available or is delivered with insufficient quality.^22,23^ Available evidence has emphasised the importance of post-abortion contraceptive counselling on uptake. In a study on determinants of post-abortion contraceptive uptake in East and Southern Africa, Uganda had the highest level of reported non-receipt of post-abortion contraception at 56%, compared with 19% in Mozambique. Nulliparous women were 60% more likely to leave the facility without a method and those who had not received post-abortion contraception had four times the odds of not initiating a method post-abortion.^24^ Results from a cross-sectional study across central Uganda showed that women who had received post-abortion contraceptive counselling had 59% higher uptake, compared to women who did not, and that multiparous women had 13% higher uptake than women with previous births. Those who reported taking up a method were most likely to choose implants or injectables.^25^

Apart from issues such as stock-outs, training needs and time constraints impacting uptake rates,^26^ qualitative research suggests that societal sexual and reproductive norms and provider attitudes influence who is offered post-abortion contraceptive counselling, what method is offered,^27^ and how counselling is provided.^20^ This may explain why some groups, such as young or nulliparous women, appear more likely to leave without a method or opt for user-dependent methods such as condoms or other short-acting methods.^25^ Meanwhile, long-acting methods, such as the intra uterine device (IUD), have several advantages over user-dependent methods, with high effectiveness and user satisfaction,^28^ and allow for concealed use. Moreover, the IUD has been identified as a key intervention in decreasing maternal mortality due to unintended pregnancy.^29^ Nevertheless, IUD use and post-abortion IUD uptake remain low in Uganda.^25,30^

While there is ample evidence on the factors hindering contraceptive use, including within the post-abortion context, in Uganda and similar settings,^7–10,27,31^ less is known about how women who initiate an IUD post-abortion navigate norms and overcome obstacles. This information may be useful for policy and practice, and specifically when tailoring PAC interventions. Nested within a randomised controlled trial comparing early versus standard timing of IUD insertion within PAC^32^ we aimed to explore perspectives on post-abortion IUD use among Ugandan women. Specifically, we wanted to understand *how post-abortion women navigated socio-cultural factors, gender norms and power relations when choosing an IUD, and how did they overcome challenges that discourage use in this context?*

## Methodology

### Setting

The Ugandan healthcare system is divided into general and regional referral hospitals and health centres of types II, III and IV, according to service provision. All public facilities aim to offer family planning services free of charge, including post-abortion contraceptive counselling, which is typically provided by midwives.^33^ Luganda is the most commonly spoken language in the central region.^34^ The study sites were four healthcare facilities, urban and rural, in central Uganda, within one to three hours drive from Kampala; one national referral hospital, one regional referral hospital, one rural general hospital and one rural health care centre. This study was part of a project that has generated several publications, including a randomised trial with the aim of increasing access to post-abortion IUDs,^32^ a cross-sectional study on post-abortion IUD uptake,^35^ and a qualitative study focusing on couples and their experiences of post-abortion IUDs.^36^ Healthcare providers who were involved in this project had received training in post-abortion contraceptive counselling with a focus on intrauterine contraception.

### Study design and respondents

In this phenomenological study, we conducted in-depth, individual interviews between 31 January and 29 August 2023 with 24 women aged 19–42 years, who had initiated IUD use after receiving treatment for an incomplete abortion (spontaneous or induced). Purposive sampling was deployed to include women with various socio-demographic characteristics and reproductive histories. We applied the concept of information power – defined as the richness and relevance of data provided by the sample – to guide our decision on recruitment conclusion.^37^ This involved continuous reflection on whether additional interviews added meaningful insights, while ensuring diversity in participants’ background characteristics, including IUD use.

Potential respondents were approached in private by a midwife while attending a post-abortion follow-up visit. The midwife provided oral and written information regarding the objectives of the study and the study procedures. The women were informed that their participation would be voluntary and that their confidentiality would be protected should they choose to participate. For those who agreed, written informed consent was obtained, the woman’s signature was witnessed and documented, and interviews were scheduled for a separate day based on the participant’s convenience. A few women declined participation, with the most frequently cited reasons being lack of time and the long travel distance to the healthcare facility. For participants aged 20 years and below, the most common reason for declining participation was their enrolment in boarding school, typically situated quite far from the health facility.

### Data collection

A semi-structured interview guide with open-ended questions was used (Annex 1). The interview guide was developed by the research team and comprised questions about the participants’ decision to initiate and continue their IUD use, including justification and motivation to use, how they overcame potential barriers, and factors that facilitated use. The guide was pilot tested prior to data collection. After three pilot interviews, the interview guide was modified to better capture the aim of the study. These three interviews were not included in the data analysis.

The first author, ELT, a female obstetrician gynaecologist from Sweden, conducted all the interviews. The remainder of the research team consisted of researchers from Sweden (AC and KGD) and Uganda (HK, OK and JB), all with extensive qualitative research experience in the Ugandan context. There were three Ugandan research assistants in the team who were familiar with qualitative research methods and the socio-cultural context of the study participants and were also fluent in Luganda. Interviews were held in an enclosed, private room where the conversations could not be overheard at the different study sites and were conducted in English or Luganda, depending on the interviewees’ language skills and preferences. For the interviews conducted in Luganda, a female Ugandan interpreter with previous experience in qualitative research was present. Before the start of the interviews, the interviewer introduced herself, her background and her role in the research project; the participants once again received oral and written information and were given opportunity to ask questions. For the first eight interviews, a female, Ugandan research assistant was present and a discussion regarding socio-cultural differences and interpretation took place after each interview. The interviews were audio recorded and lasted approximately 40 minutes.

### Data analysis

The interviews were transcribed verbatim by a research assistant fluent in both English and Luganda and then translated into English. The quality of the transcription and translation was then checked by listening to the interviews while reading the transcripts. Changes were made where necessary. The analysis followed Braun and Clarke’s “six-phase analytical process” of reflexive inductive thematic analysis, which enables openness and flexibility concerning emerging themes.^38^ First, the transcripts were read through several times, after which each transcript was coded using the software program NVivo version 14.24.0. ELT led the data analysis and performed the initial coding. The data were analysed at the latent and manifest levels and then these codes were organised into groups of codes that represented different aspects of the data. The groups of codes were then organised into initial themes, and subsequently synthesised into interpretive sub-themes and themes, which represented meaningful and broader patterns that we identified in the data. The process of coding and organising the data into themes, refining themes and their interpretation were discussed iteratively between ELT and AC and then with the full research team. Data analysis occurred in parallel with data collection.

Each step of the research process was documented thoroughly. No repeat interviews were held, but one respondent was contacted again for a minor clarification. In cases of uncertainties during the analysis process, the interpretation of the findings was discussed within the research team, including both Ugandan and Swedish researchers. ELT also employed reflexive journaling where she documented personal thoughts, observations from the interviews and reflections throughout the study that she discussed with the research team.

### Ethical considerations

Ethical clearance was granted by the Makerere University School of Medicine Research and Ethics Committee (reference number 2021-131, approved 31st January 2022 until 31st January 2025) and Uganda National Council for Science and Technology (reference number HS2111ES, approved 10th May 2022 until 10th May 2026). Ethical clearance for the analyses conducted in Sweden using data stored there was provided in advance by the Swedish Ethical Review Authority Board on 3 April 2023 (reference number 2023-01263-01, valid until 31st December 2024). All participants received monetary compensation to cover the cost of transportation. To protect the identity of the participants, the data were anonymised and stored safely in a password-protected computer.

## Findings

The respondents comprised 24 women between the ages 19 and 42, who were residing in urban, rural or semi-urban areas in and around Kampala (Table 1). All respondents but one were married or in a relationship and the majority had given birth at least once. Their education levels varied from incomplete primary school level to completed university education. Some had used modern contraceptive methods before, most commonly the pill, implants, injectables and/or emergency contraception. For others, the IUD was their first method of modern contraception. Respondents had chosen the IUD because it had fewer side effects, was safe and long acting, had a non-hormonal option and was possible to use covertly. Three women had discontinued their IUD, two because they wanted to become pregnant and one because their IUD was expelled.

**Table 1.** Background characteristics of the participants

Background characteristics	*N* = 24
**Age**	
15–25	5
26–35	12
36–42	7
**Area of residence**	
Urban or semi-urban	16
Rural	8
**Education (complete or incomplete)**	
Primary education	5
Secondary education	12
Post-secondary/university	7
**Marital status**	
Married	19
Unmarried	5
**Parity**	
0	4
1–2	9
3–5	11
**Currently using an IUD**	
Yes	21
No	3
**Concealed IUD use**	
Yes	8
No	16

**Figure 1.**
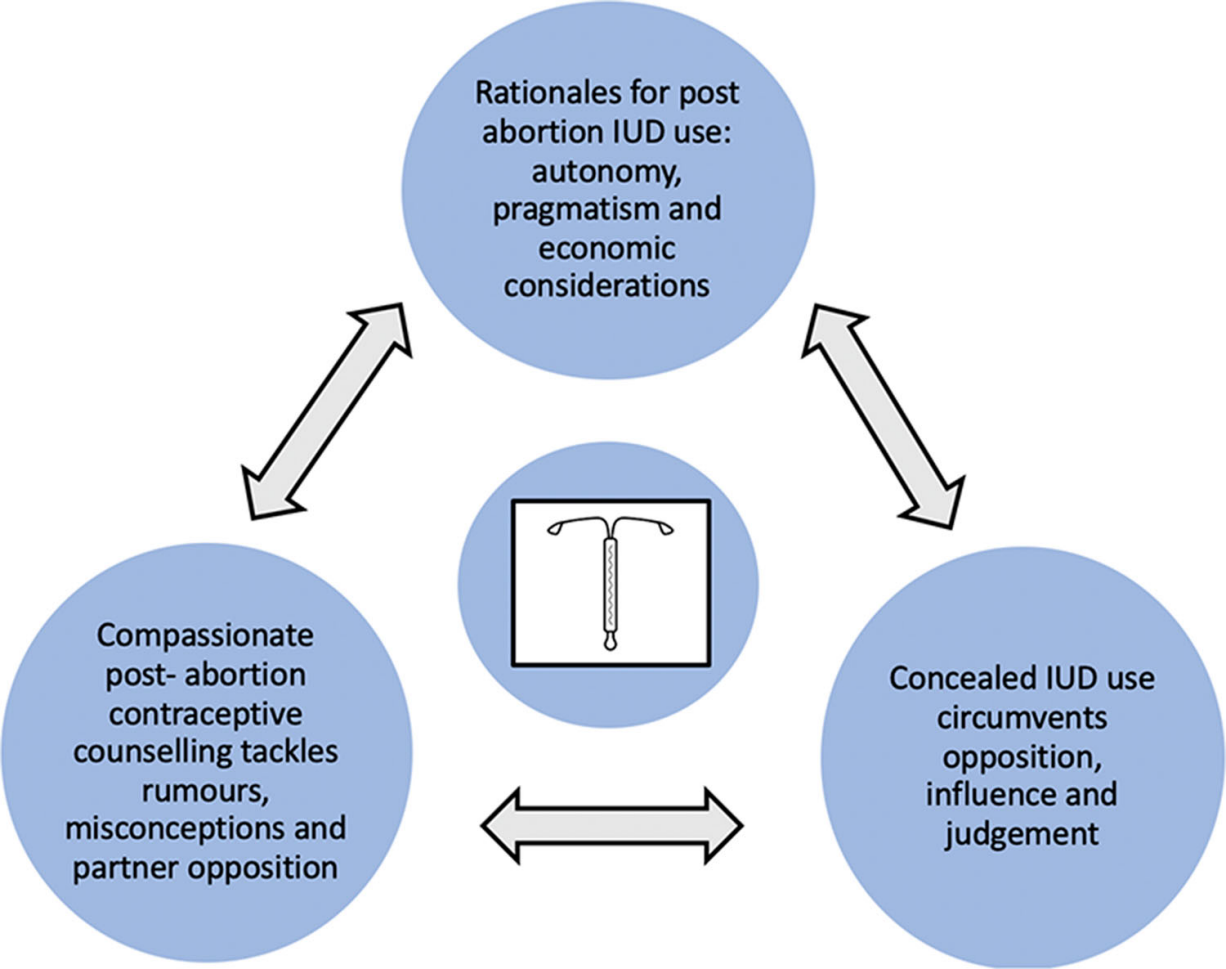
Theme matrix

Three themes were constructed in the analysis process: (1) rationales for post-abortion IUD use: autonomy, pragmatism and economic considerations, (2) compassionate post-abortion contraceptive counselling tackling rumours, misconceptions and partner opposition, and (3) concealed IUD use circumvents opposition, influence and judgement. The three themes and their interlinkages are illustrated in Figure 1.

Respondents relayed various reasons for initiating an IUD, using a combination of interlinked arguments around pragmatism, economic factors and autonomy. These arguments were grounded in social and gender norms surrounding fertility, sexuality, and gender roles in society and in the home, and in the respondents’ personal circumstances. Most respondents declared that it was themselves who independently made decisions around reproductive choices and contraceptive use in their relationship or family. Still the pressure of traditional norms, financial constraints, and expectations on fecundity and large family sizes, were evident in their testimonies and meant that to use an IUD they had to circumvent the influence of peers, partners, parents and in-laws. Accessing contraceptive counselling and concealing IUD use emerged as key strategies to achieving their reproductive goals. Moreover, these two strategies also helped support the respondents in their decision to use an IUD by receiving information and support, through contraceptive counselling, and by enabling them to disregard other people´s opinions in favour of their wishes and needs, through covert IUD use.

### Rationales for post-abortion IUD use: autonomy, pragmatism and economic considerations

Respondents of all ages and backgrounds used a combination of arguments to explain their decision to use an IUD. Arguments centred around the right to health and bodily integrity were interlaced with pragmatism around the risk of being abandoned, financial constraints, and expectations around motherhood and women’s responsibility for children in Uganda. Further, economic constraints were used to argue for smaller family sizes even when this went against traditional reproductive norms. Respondents spoke about pregnancy and childbirth as experiences that affected women’s bodies and lives and therefore, within a couple, women were entitled to make decisions related to these matters. Pregnancy and childbirth, and particularly short birth intervals, were described as fraught with biomedical risks, with negative consequences for their health and for the family, necessitating contraceptive use, as described below:

*“Giving birth normally affects the lady and not the men, so I think we need to stand firm on using family planning*.*”* (Teacher, 41 years old, five children)

*“If we (me and my husband) have different opinions, I think it will be me to decide because it’s me to become pregnant, to carry the pregnancy for nine months and also face the risks. I will be the one to decide on how many I will be able to give him.”* (Unemployed, 19 years old, no children)

By using phrases such as “nowadays” or “these days,” many participants claimed that attitudes towards reproductive decision-making were changing towards restricting the number of children and greater autonomy for women, as exemplified by one of the respondents:

*“These days it’s the women who make the decisions, so they don’t care if they [husbands or in-laws] say yes or no. Things are changing, things are modernizing*.*”* (Miner, 31 years old, two children)

Many respondents expressed dissatisfactions with how other people (e.g. in-laws, church leaders and family) interfered in their reproductive decision-making, despite the burden of responsibility for the children being theirs alone to carry. Sentiments of entitlement to make reproductive health decisions independently were expressed by a majority of the participants, regardless of education level, marital status, parity, religion or occupation.

*“For me, I decided for myself. I left whatever beliefs my society and my friends had. I decided to take it (the IUD) depending on my life situation. Whatever they said about it couldn’t deny me for getting what I wanted.”* (Student, 26 years old, no children)

*“It´s me to decide because I am the one who know the situation that I am going through.”* (Farmer, 28 years old, two children)

However, some respondents expressed the need to ask permission from their husband, or at least discuss it with him, before going ahead with the IUD insertion.

*“Yes, of course, I had to first talk to him and if he had refused then I couldn’t opt for it. But fortunately, he didn’t refuse, he just said go ahead with it.”* (Businesswoman, 34 years old, 3 children)

However, a few respondents expressed that while they supported the right of women to independently make their own reproductive decisions, it was their partner who had the final say in their personal relationship.

A smaller family size was also described as more practical given the fact that women were expected to raise and care for the children, and there were no guarantees that the husband or the in-laws would live up to their responsibilities.

*“These days, in-laws like to have kids, but they don’t take responsibility for them. They will pressure you to get pregnant, but they won’t be around when you are suffering to raise those children.”* (Unemployed, 19 years old, no children)

Respondents spoke about a general pattern in society where male partners’ extramarital affairs and lack of or unpredictable support influenced women’s use of contraceptives. Some respondents therefore planned for the possibility that their partner would abandon them and planned their childbearing and contraceptive use around their own finances to make sure that they would be able to take care of the children once single.

*“I want three children based on my financial status. Even if the man leaves tomorrow, I will manage taking care of the 3 children*.*”* (Unemployed, 22 years old, one child)

*“You are put under pressure that you produce many children for the clan or family, and after that, when the husband goes away, you are left alone to take care of all the kids.”* (Unemployed, 19 years old, no children)

Respondents emphasised the significance of contraception and how it supported them to plan when to become pregnant and space between pregnancies. This mindset was described as increasingly common in society and especially important in urban areas, where costs related to housing, school fees and food were increasing, as described by one respondent:

*“What I liked (with the IUD) is that it helps you to get time, it helps you to space and you are able to prepare for the kids. I don’t want to add any other kids. I want us to plan for the two that we have.”* (Farmer, 42 years old, 2 children)

For some respondents, planning childbearing around the family’s financial situation was done with mutual understanding within couples. If finances improved, the couple could go ahead and conceive another child, as explained by one married participant:

*“Yes, because now we (my husband and I) think that even the children we have we feel they are just enough. Probably if the economy becomes okay, we can remove it (the IUD) and get more but not now. It will be a planned pregnancy. Not this abrupt pregnancy when you have not planned for it, no.”* (Social worker, 34 years old, four children)

### Compassionate post-abortion contraceptive counselling tackles rumours, misconceptions and partner opposition

Respondents talked about how good-quality contraceptive counselling had been important for their ability to initiate and continue with their IUD use. Contraceptive counselling from a trained provider made them more confident in their decision-making, challenged rumours and misconceptions around the IUD, enabled concealed use and helped them convince their partners. Being advised by someone they trusted was mentioned by several participants as important in supporting their decision to use an IUD. Some respondents spoke about how the compassion shown by providers during their hospital stay receiving post-abortion care made them feel safe to use the IUD and more confident to make this decision independent from their partners:

*“I was so ashamed and shy. But she (the midwife) told me you are going to calm down and I am going to help you. (…) It was the information she gave me that made me feel comfortable. She said that when you feel like you want a child you just come back. And I thought that I might just complete my studies first.”* (Student, 26 years old, no children)

The contraceptive counselling also played a crucial role in contradicting rumours and misconceptions. Several respondents expressed that the information they received from the provider contradicted what the participants had previously heard about IUD use from family, friends or the community, as explained by this mother of three below:

*“They (people in the community) were saying that it’s not good (the IUD), that sometimes it can go inside, it can even disappear. some even say it’s cancerous. So it’s the way she (the health worker) talked to me about it. She gives you good information and you get to understand that people are just talking bad things about it yet they don’t know it exactly.”* (Business woman, 34 years old, 3 children)

Contraceptive counselling was also a means in getting their partner to agree with their IUD use. Once home from the hospital, respondents re-cited the information provided to them at the counselling session to their partner, including information about benefits and side effects of the IUD, addressing common rumours and misconceptions. This information confronted common fears and concerns and made the respondents and their partners feel safe and encouraged.

*“Yes, he was ok with it because when I was told the side effects and benefits, I went back and explained everything to him, so I gave him all the alternatives and he was like we can go by the other one (the IUD).”* (Businesswoman, 33 years old, with one child)

*“The fact that I explained to him everything that health workers taught me, he didn’t get worried.”* (Shop owner, 42 years old, with 3 children)

A few respondents had brought their partner back to the healthcare clinic for them to receive contraceptive counselling together. Others told how contraceptive counselling supported their continued IUD use. One respondent contacted a health worker to receive additional information when concerns arose:

*“At first, he didn’t mind about it (the IUD) but later he told me that he was feeling it. When I talked to the health worker here, they told me that it would get better and he won’t feel it with time.”* (Unemployed, 19 years old, no children)

Spreading information about and organising counselling sessions on contraception was also described as an important intervention in improving contraceptive use in Uganda, both in terms of providing accurate information but also to support women in their decision-making and autonomy.

*So, I think that they (women) need to be more sensitised about family planning. They need to know that it’s them to decide, not their husbands, because it’s their lives which is at risk. With this sensitisation, women can have that confidence within them that they can stand and say, “I am using family planning whether my husband wants or not.”* (Unemployed, 19 years old, no children)

### Concealed IUD use circumvents opposition, influence and judgement

The fact that the IUD could be used covertly was what made it attractive to some respondents. Covert use enabled attainment of reproductive goals while circumventing partner opposition and the influence of others, such as peers and in-laws, and providing protection from rumours and judgement by other community members. Partner refusal, either evident or anticipated, was commonly discussed among respondents with both covert and open use, as a reason for secrecy. Misconceptions, fear of side effects and a wish for more children were cited as the most common reasons for partner opposition to contraceptive use. Regardless of whether a respondent had a partner who was aware of their IUD use, many stressed that contraceptive use was a private matter. To prevent rumours or unsolicited opinions from friends and family, they preferred to keep their use hidden.

*“There were some people in the university talking about family planning and that you can go to Kawempe. I heard her say I can’t even risk myself to go there. The IUD; it would just damage your uterus and you lose your pregnancy. By that time, I already had it there. So, I decided to keep quiet and let them say a lot of things. I don’t need to share any of this information.”* (Student, 26 years old, no children)

In contrast, a few respondents shared that influence played a key role in their decision to use the IUD, with friends serving as valuable sources of support and information.

*“Yes, because I heard from her (the friend).. So, I also opted for it (the IUD) since I had never used any family planning method. So, I had to go for that one that she wanted. It was like that … So, friends are also great influencers.”* (Businesswoman, 34 years old, 3 children)

Nevertheless, by keeping the IUD use a secret from friends and family, even if the partner was aware of it, covert use prevented community influences from reaching and affecting the partner.

*“The truth is using an IUD is a private matter, I cannot tell you that this one uses it or not, because no one wants anyone to decide for you. You just go with the husband and you do it alone because if people get to know about it, they can influence your husband to leave you. This is a secret thing.”* (Farmer, 42 years old, 2 children)

One respondent explained how covert use could be used to alleviate pressure from society to have more children.

*“The reason why people keep it a secret is because some religions say you are killing children, so a person keeps it a secret, she and the doctor are the ones who know about it … Also, people keep it a secret because once society gets to know, they look at you as a disgrace to society because you are using family planning and you are not having the large family as expected”*. (Selling vegetables, 35 years old, 4 children)

Covert IUD use thus enabled the respondents to appear to adhere to traditional reproductive norms while in fact tending to their own wishes and needs. However, this strategy did not seem entirely uncomplicated, since some participants were hesitant to admit to being secretive with their partners. Further, several participants expressed feelings of resentment and anger over men’s strong influence and the need for secrecy.

*“It’s not good what men do, to tell women not to use family planning, to decide for them. These women should stand up for themselves and make the decision.”* (Unemployed, 22 years old, one child)

## Discussion

This qualitative study explored how post-abortion women in Uganda with current or recent IUD use navigated socio-cultural factors, gender norms and power relations, and overcame challenges that hinder contraceptive use. Our findings show how justifications for post-abortion IUD use, be it overt or covert, were shaped by social perceptions around gender roles and responsibilities, as well as personal circumstances including economy and available partner support. Within this context, women seemed to view reproductive autonomy as their prerogative. Compassionate empathetic post-abortion contraceptive counselling and concealed IUD use enabled women to achieve their reproductive goals despite individual and contextual impediments. These findings highlight the importance of quality post-abortion contraceptive services in supporting women’s decision-making and reproductive choices.

In the context of prevailing gender norms and personal economic and relationship circumstances, women in our study expressed their right to make autonomous decisions about their reproductive health. These sentiments were shared by respondents across urban and rural areas, spanning diverse ages, educational backgrounds, and professions. Similar findings have been reported by Kibira et al^39^ from Nigeria, Uganda and Ethiopia, showing that both male and female respondents in all three settings considered decision-making related to contraception (in general) to be in the domain of women’s responsibility and rights. Similarly, a study from Mozambique on empowerment for reproductive decision-making, but not within the PAC setting, showed that women felt entitled to make reproductive health decisions.^40^ Nevertheless, it remains unclear how our respondents’ experiences of a spontaneous or induced abortion and related care-seeking shaped their sense of bodily autonomy and whether similar arguments exist in other PAC settings or other health-related decisions, in Uganda.

Women’s empowerment is connected to employment status, education levels, socioeconomic status and involvement in domestic decision-making and its significance for reproductive health and well-being is well documented.^3,41–44^ Research suggests that women’s empowerment in East Africa has improved between 1995 and 2015^41^ and increasingly so for every generation.^42^ However, significant challenges remain; many women’s decision-making is constrained by a complex web of socio-cultural factors that limit their decision-making autonomy.^31^ In our study, some respondents advocated for their own and other women’s rights to make reproductive health decisions, yet they appeared to have limited agency within their intimate relationships. This highlights a disconnect between the recognition of empowerment and the actual realisation of it in personal contexts.

Although further studies are needed to explore this finding, it suggests that discussions around bodily rights and integrity within the PAC setting, may be helpful in interventions aimed at improving access to and use of contraception.

Our findings show how relationship instability – including the calculated risk of becoming a single mother – was alluded to as a major reason for women to opt for post-abortion IUD use, a finding supported by other studies on contraceptives from Uganda and East Africa.^10,39,44,45^ While negative impacts of gender inequality on contraceptive use in Uganda have been documented previously,^9,10,46^ our findings suggest an awareness of these norms and expectations, and illustrate how women actively navigate them and the unequal power dynamics that characterise their realities. Initiatives targeting patriarchal norms and gender power imbalances have been associated with increased gender equality and contraceptive use.^47^ As such, gender-transformative initiatives that challenge harmful norms and unequal power dynamics are needed to move Uganda closer to achieving Sustainable Development Goal 5 regarding gender equality.^48^

Our interviews highlight the importance of good quality post-abortion contraceptive counselling. Although we did not assess the quality of post-abortion counselling, respondents described interactions with providers that were compassionate and supportive, which in turn is known to increase contraceptive use and satisfaction with chosen methods.^49^

One-third of our participants used their IUDs covertly, which allowed them to control their fertility and lives, in line with previous studies from this setting showing how covert use helped women limit family size while fulfilling their responsibilities as women in their families and societies.^10,44,46,50^ A recent report from the Ugandan Ministry of Health^4^ showed that 20% of Ugandan women who used a modern contraceptive method concealed their use from their partner. While most participants in our study cited their reason for covert use as partner refusal, some expressed that they had not really discussed the matter with their partner. Poor (or no) couple communication may contribute to covert use,^44^ which in turn has been associated with increased suspicion, threats and male partner violence,^39^ Still, gendered expectations that assign men authority and women domestic responsibility, alongside power imbalances and discordant views on childbearing within couples,^51^ propel women towards this practice.

It is, therefore, imperative that healthcare providers engaged in post-abortion contraceptive counselling are aware of the risk of violence^4^ and can advise women on suitable methods that are less likely to be detected, thereby respecting and supporting their choice and autonomy. Future research should focus on how to best integrate safety planning in post-abortion contraceptive services to support women at risk of violence and help them meet their reproductive needs.

The Ugandan government has committed to increase and improve contraceptive initiatives and access and recognises the importance of contraceptives in economic development.^4^ Our findings emphasise the importance of prompt and continuous efforts to secure access to evidence-based post-abortion contraceptive counselling in Uganda that supports overt and covert use, coupled with empowerment-focused and gender-transformative initiatives targeting harmful norms and gender power imbalances. Our findings may be useful to PAC providers whose support for women’s needs and preferences are key to strengthening sexual and reproductive health and rights in Uganda.

### Limitations

As this study was conducted among post-abortion women who had used IUDs, it is possible that the opinions expressed in the current context were more positive towards IUD use than among women with no IUD experience. The study’s findings reflect a specific group of women who accepted an IUD after receiving post-abortion contraceptive counselling within a controlled trial setting. As contraceptive counselling was delivered under atypical conditions, the results may not be directly transferable to other Ugandan clinical contexts, or settings where post-abortion counselling is limited or of poor quality.

In our efforts to counteract social desirability, we built rapport with study participants and emphasised that there were no right or wrong answers. Still, we cannot be certain that social desirability has not impacted our findings. Moreover, because ELT is not a Ugandan native, her perspective as a foreigner, with a different socio-cultural background, could potentially affect the findings and their interpretation. To minimise this risk, the findings were discussed iteratively within the research team consisting of both Ugandan and Swedish researchers. Lastly, Uganda comprises over 40 tribes, each with different languages and cultures.^34^ This study was conducted in the central region of Uganda, predominantly occupied by the Baganda people; therefore, our findings might not be transferable to other parts of Uganda.

## Conclusion

Pragmatism, personal circumstances and the sense of reproductive autonomy are important considerations for women in their post-abortion reproductive planning that shape covert and overt IUD use. Post-abortion contraceptive counselling plays an important role in supporting women in their decision-making to achieve their reproductive goals while circumventing obstacles. Efforts that ensure access to good-quality post-abortion contraceptive counselling coupled with gender-transformative initiatives are therefore needed to strengthen the sexual and reproductive health and rights of women and girls in Uganda.

## Supplementary Material

Interview guide: Womens perceptions on post-abortion contraception

## Data Availability

The datasets used and/or analysed in the current study are available from the corresponding author upon reasonable request.
