# Supplementary material for: Balancing pragmatism, norms and power relations: a qualitative study among post-abortion intrauterine device users in central Uganda
Source: Sex Reprod Health Matters. 2026 Jan 2;33(1):2604886. doi: 10.1080/26410397.2025.2604886 (PMC12825586; doi:10.1080/26410397.2025.2604886)
Supplement: Interview guide: Womens perceptions on post-abortion contraception [file ZRHM_A_2604886_SM0803.docx]

Women’s perceptions on post abortion contraception, especially the IUD

# Interview guide

**Aim**

The aim of this study is to explore perspectives on post abortion contraceptive uptake and use among Ugandan women. Specifically, we want to gain a deeper understanding of how women with current or recent IUD use, navigate socio-cultural factors, gender norms and power relations and overcame challenges that discourage use in this context.

**Research questions**

- What are women’s perceptions surrounding post abortion contraceptives and the IUD in particular?
- Considering the many socio-cultural believes and practices, gender norms and power relations that discourage modern contraceptive use, how do women who have chosen to initiate mPAC (post abortion care with medical treatment) IUD assert their agency, negotiate use and overcome obstacles to use?
- How do current social and gender norms including norms about sexuality and reproduction influence reproductive agency in relation to contraceptive use?

**Interview guide**

1. **Can you please tell me a little about yourself?**

- Age
- Education
- Occupation
- Marital status (polygamous union)?
- Parity
- Rural or urban residence
- Previous experience of modern or traditional contraceptives?
- Religion

1. **Can you please tell me how you came to choose to initiate the IUD and your experiences with it so far?**

- What made you want to use a modern method at this time in your life?
- Post miscarriage or induced abortion?
- What influenced the decision?
- What made you accept this particular method?
- Is your partner aware of that you are using an IUD? Does he agree with this decision?

*(Probe on issues of acceptability here and satisfaction with the method. If she has discontinued find out why)*

**Can you tell me your thoughts on the use of modern contraceptives in Uganda in general?**

- Is it common for women to use contraceptives? What influences use? Barriers/facilitators to use?
- Barriers/facilitators to use of the IUD in particular?
- *How did any of these barriers or facilitators affect your decision when choosing the IUD?*

**What are your thoughts on traditional socio-cultural norms in Uganda that promote large families and frequent childbirth?**

- Do you think these norms influence Ugandan women’s reproductive choices?
- Did it have a role in your own decision?

**What are your thoughts on more modern norms that promote limiting the number of children you have, delay childbearing and practice spacing?**

- How does this norm fit into Ugandan culture?
- Did it have any role in your own decision?

**Can you tell me about your thoughts on childbearing and your plans for the future?**

- Do you and your partner agree? If not, who will make the final decision? Do your in-laws agree? Do they have a say?

**What do you think is needed in order for more women in Uganda to use modern family planning methods such as the IUD?**
